# Sex and race define the effects of adverse childhood experiences on self-reported BMI and metabolic health biomarkers

**DOI:** 10.1186/s13293-022-00439-x

**Published:** 2022-06-15

**Authors:** Jacqueline R. Leachman, Kory Heier, Feitong Lei, Nermin Ahmed, Carolina Dalmasso, Meredith S. Duncan, Analia S. Loria

**Affiliations:** 1 Department of Pharmacology and Nutritional Sciences, University of Kentucky, Lexington, KY 40536-0200 USA; 2 Department of Biostatistics, University of Kentucky, Lexington, KY USA; 3 Center for Health Equity Transformation, University of Kentucky, Lexington, USA

**Keywords:** ACE, BMI, Waist circumference, Race, Ethnicity, Sex, Metabolic risk

## Abstract

**Background:**

Adverse childhood experiences (ACEs) are an independent risk factor for chronic diseases, including type 2 diabetes, stroke and ischemic heart disease. However, the effect of ACEs considering sex and race are not often reported in cohorts showing multiracial composition, with power to evaluate effects on underrepresented populations.

**Aim:**

To determine how sex and race affected the association of combined and individual ACEs with metabolic health biomarkers in the Southern Community Cohort Study (2012–2015).

**Methods:**

Self-reported data were analyzed from ACE surveys performed during the second follow-up of a cohort comprised by over 60% of Black subjects and with an overall mean age of 60 years.

**Results:**

BMI steadily increased with cumulative ACEs among Black and White women, but remained relatively stable in White men with ≥ 4 ACEs. Contrary, Black men showed an inverse association between ACE and BMI. Secondary analysis of metabolic outcomes showed that physical abuse was correlated with a 4.85 cm increase in waist circumference in Black subjects. Total cholesterol increased among individuals with more than 4 ACEs. In addition, increases in HbA1c were associated with emotional and maternal abuse in Black women and sexual abuse in White women.

**Conclusions:**

BMI is strongly associated with cumulative ACEs in women regardless the race, while waist circumference is strongly associated with ACEs in Black individuals, which combined with reduced BMI may indicate increased central adiposity in Black men. Our study suggests that sex and race influence the contribution of certain ACEs to impair metabolic health.

**Supplementary Information:**

The online version contains supplementary material available at 10.1186/s13293-022-00439-x.

## Highlights

- This study shows the effects of ACEs in a cohort with a unique multiracial composition and enough power to evaluate these effects in underrepresented populations.
- Body adiposity is strongly associated with cumulative ACEs in women regardless of race.
- Only Black men displayed a negative correlation between cumulative ACEs and body adiposity. However, Black individuals showed an increased waist circumference with physical abuse suggesting an effect of ACEs on increased central adiposity and metabolic risk in this population.
- Specific adverse experiences seem to alter metabolic biomarkers with a unique signature depending on sex or race. Further investigation of these associations is critical to developing a more effective preventive and personalized medicine to reduce health disparities.

**Supplementary Information:**

The online version contains supplementary material available at 10.1186/s13293-022-00439-x.

## Introduction

The obesity epidemic in the United States of America affects 42.4% of the population regardless of sex; however, age adjustments show that severe obesity is 9.2% higher in women [1]. Additionally, the current statistics project that 50% of adults will have obesity by 2030 [2]. Adults with obesity typically have multiple organ system complications and, as a result, are at higher risk for heart disease, stroke, type 2 diabetes, and multiple types of cancers [3–6]. An important limiting factor in controlling this epidemic is related to the unsuccessful long-term results in the clinical management of obesity [7, 8]. As there is a need for further investigation into the potential causes that may dampen the effectivity of treatments to prevent or reduce obesity, identifying modifiable risk factors affecting men and women’s obesity trajectories becomes a key step to addressing health disparities.

The development of obesity is a complex process influenced by genetics and environmental factors. Lifestyle, nutrition, psychosocial environment, and socioeconomic status have been shown to shape the well-being of adult individuals, showing extremely detrimental consequences when occurring during the first decade of life. Notably, childhood obesity and the psychosocial influence of racial and ethnic disparities at a young age may have an unforeseen contribution to the obesity epidemic [9, 10]. Current growth trajectories predict that over half of toddlers and children will be obese by the age of 35 [11]. Supporting these trajectories, clinical and experimental studies have shown that prenatal life and early childhood determine the predisposition of the individual to gain weight and/or develop impairments in energy metabolism homeostasis [12, 13]. Therefore, any early life event that prevents the development of positive nutritional and lifestyle habits during childhood, including the influence of caregivers modeling healthy behaviors, may have a life-long deleterious impact on weight trajectories [14–16].

Large cohort studies have provided evidence showing that adverse childhood experiences (ACEs) are strongly associated with increased body mass index (BMI) and insulin resistance later in life [17–21]. While it is well documented that women show increased rates of stress-related comorbidities such as anxiety, depression, obesity, and ischemic heart disease during adult life [22–24], less is known about the effects of ACE on their cardiometabolic function [21, 25–27]. The scientific literature describing interventions for the treatment of obesity or overweight women with a history of ACE is limited [28]. Furthermore, results from the Americans’ Changing Lives study revealed a direct association between exposure to ACEs and higher levels of adulthood stress in both men and women; however, women with high number of ACEs gained weight more rapidly, while no association between BMI and ACE was found in men.

The Southern Community Cohort Study (SCCS) is a prospective cohort established in 2001 to address the origins of health disparities on chronic diseases [29]. Using a part of this foundational cohort that responded to the ACE questionnaire in a second follow-up, Mouton et al. reported that over half of all SCCS subjects experienced at least one ACE, with a greater prevalence in women and Black subjects. Moreover, this study revealed that ACEs are associated with adult cancer risk behaviors, such as smoking, and among women, lower mammography and Pap screening rates [30]. Furthermore, this study also identified that ACEs significantly increased the odds ratio of developing morbid obesity in White people and Women. However, the analysis was not stratified by sex and race. Since non-Hispanic Black and women are well represented in the SCCS, we designed the current study to determine how sex and race affected the association of combined or individual ACEs with self-reported BMI and metabolic health biomarkers available in a senior population.

## Methods

### Study sample

The individuals contained in the SCCS are primarily African American and non-Hispanic White participants recruited from Alabama, Arkansas, Florida, Georgia, Kentucky, Louisiana, Mississippi, North Carolina, South Carolina, Tennessee, Virginia, and West Virginia. The SCCS was approved by the Vanderbilt University Medical Center Institutional Review Board; analysis data were deidentified and did not require local IRB approval.

This sample included a subset of 41,248 participants who participated in the second follow-up ACE questionnaire. We limited our analysis to non-Hispanic Black and White men and women (*n* = 39,150) since there were few participants of other race and ethnicity to allow group stratification. We additionally excluded participants missing 4 or more ACE questionnaire components (*n* = 797). Thus, our final sample size for analysis included 38,353 non-Hispanic Black and White men and women.

### Outcome

Our primary dependent variable was BMI, defined as self-reported weight in kilograms divided by height in meters squared. Secondary dependent variables were waist circumference (self-reported in cm) and blood biomarker measurements including leptin, adiponectin, total cholesterol, high-density lipoprotein (HDL) cholesterol, C-peptide, HbA1c, insulin, and resistin. Briefly, a 20 mL venous blood sample (10 mL drawn into an EDTA vacutainer tube, and 10 mL drawn into a serum vacutainer tube) was collected. Blood samples were spun at 1500×*g* for 10 min, using a refrigerated centrifuge (at 4 °C). The plasma was then removed and pipetted into four sterile 2 mL cryovials. After collection, the blood samples were kept refrigerated, and at the end of each day packed in a Styrofoam box with ice packs and sent via Federal Express for next morning delivery to the Vanderbilt Ingram Cancer Center for processing. The blood samples were typically processed within 4 h of arriving at the laboratory. All samples are stored in appropriate freezer boxes and kept at − 80 °C.

### Exposure

Upon enrollment, the questionnaire was administered during assisted personal interviews at community health centers or via self-completed questionnaires for the general population sample. The SCCS questionnaire at the second follow-up included ten questions regarding ACE exposure. An individual’s response to each ACE item was recorded as yes or no. A person’s total ACE score ranged from 0 to 10 and was calculated as the sum of all “yes” responses. The list of questions is provided in the Additional file 1.

### Covariates

Self-reported age in years, sex (male/female), race (non-Hispanic Black or White), and pre-/post-menopausal status served as covariates.

### Missing data

For this investigation, we used multivariate imputation by chained equation technique that generated ten complete datasets to handle missing covariate data while maintaining the correlation structure. In addition, regression-based predictive mean matching was utilized to produce biologically plausible imputed values. Results across imputed datasets were combined according to Rubin’s rules.

### Statistical analyses

Summary statistics were calculated overall and by sex; continuous variables were reported as mean (standard deviation [SD]) and median (interquartile range [Q1, Q3]), while categorical variables were reported as number and percentage per category. We then sought to examine the association between ACEs and BMI using a series of linear regression analyses. First, using partial F-tests, we assessed whether there was a nonlinear association between BMI and ACEs differed by sex and race or by menopause status in women. Then, we modeled the total ACE score using restricted cubic splines with five knots and included interaction terms between menopause status (women only), sex, and race, with adjusting for age. When the interaction between sex, race, and ACE was significant, additional analyses were stratified by sex and race. We then created race and sex-specific models to assess the association between individual ACE components and BMI, adjusting for age and menopause status (women only).

The regression analyses were repeated for each of: waist circumference (*n* = 4815), total cholesterol (*n* = 2548), high-density lipoprotein (HDL) cholesterol (*n* = 2548), leptin (*n* = 805), adiponectin (*n* = 2395), C-peptide (*n* = 2394), HbA1c (*n* = 1219), insulin (*n* = 446), and resistin (*n* = 333) serving as the dependent variable (separate models for each; sample size available for each analysis provided in parentheses). The composition of groups for each secondary analysis is provided in Additional file 1: Table S1.

In the absence of a nonlinear association between ACEs and the dependent variable, the association was modeled with a simple linear term, and the beta coefficient for the increase in the dependent variable per additional ACE was reported rather than using restricted cubic splines. In the absence of significant interactions by sex and/or race, results were aggregated and adjusted for sex and race (rather than stratified by them) both when assessing the effect of the total ACE score and each ACE component separately. All analyses were completed in R version 4.1.1. A two-sided *p*-value < 0.05 was considered statistically significant.

## Results

### Sample characteristics

The sample for this study comprised 38,353 individuals, 66% of whom were female, and 63% were Black (Table 1). ACEs were more prevalent among women than men. The average BMI was in the obese range (mean BMI = 31.9 kg/m^2^) among women and was in the overweight range (mean BMI = 28.7 kg/m^2^) among men. In addition, 94% of women (*n* = 23,847) were post-menopausal.

**Table 1:** Sample characteristics by sex

	Women (*N* = 25,487)	Men (*N* = 12,866)	Overall (*N* = 38,353)
Age, years			
Mean (SD)	60.5 (8.66)	60.8 (8.38)	60.6 (8.57)
Median [Q1, Q3]	59.0 [54.0, 66.0]	60.0 [54.0, 66.0]	60.0 [54.0, 66.0]
Race			
White	9079 (35.6%)	4974 (38.7%)	14,053 (36.6%)
Black	16,408 (64.4%)	7892 (61.3%)	24,300 (63.4%)
Income*			
Less than $15,000	11,669 (49.7%)	5076 (41.9%)	16,745 (47.0%)
$15,000 to < $25,000	5075 (21.6%)	2191 (18.1%)	7266 (20.4%)
$25,000 to < $50,000	3831 (16.3%)	2185 (18.0%)	6016 (16.9%)
$50,000 to < $100,000	2170 (9.24%)	1802 (14.9%)	3972 (11.2%)
$100,000+	741 (3.16%)	869 (7.17%)	1610 (4.52%)
Marital status*			
Married/living with partner	8036 (32.1%)	6376 (50.4%)	14,412 (38.2%)
Separated/divorced	7916 (31.6%)	3126 (24.7%)	11,042 (29.3%)
Widowed	4973 (19.8%)	732 (5.78%)	5705 (15.1%)
Single	4130 (16.5%)	2420 (19.1%)	6550 (17.4%)
Menopause status			
Pre-menopausal	1541 (6.07%)	–	1541 (6.07%)
Post-menopausal	23,847 (93.9%)	–	23,847 (93.9%)
Employment status*			
Full-time	5110 (20.8%)	2661 (21.3%)	7771 (21.0%)
Part-time	1998 (8.14%)	787 (6.31%)	2785 (7.52%)
Unemployed	2456 (10.0%)	1394 (11.2%)	3850 (10.4%)
On disability	8084 (32.9%)	4140 (33.2%)	12,224 (33.0%)
Retired	5636 (23.0%)	3487 (28.0%)	9123 (24.6%)
Housewife	1267 (5.16%)	0 (0%)	1267 (3.42%)
Number of ACEs			
Mean (SD)	1.75 (2.27)	1.43 (2.07)	1.64 (2.21)
Median [Q1, Q3]	1.00 [0, 3.0]	1.00 [0, 2.0]	1.00 [0, 2.0]
BMI, kg/m^2^			
Mean (SD)	31.9 (8.01)	28.7 (6.32)	30.8 (7.63)
Median [Q1, Q3]	30.7 [26.16, 36.28]	27.7 [24.41, 31.87]	29.5 [25.45, 34.77]
Waist circumference, cm*			
Mean (SD)	104 (18.5)	103 (17.8)	104 (18.3)
Median [Q1, Q3]	103 [92, 116]	101 [92, 113]	102 [92, 115]
Total cholesterol, mg/dL*			
Mean (SD)	208 (44.1)	189 (40.6)	203 (44.0)
Median [Q1, Q3]	203 [178, 233]	187 [160.0, 214.5]	199 [173, 228]
HDL cholesterol, mg/dL*			
Mean (SD)	51.7 (14.5)	44.9 (14.6)	50.1 (14.8)
Median [Q1, Q3]	50.0 [42, 59]	42.0 [35, 52]	48.0 [40, 57.62]
Leptin, ng/mL*			
Mean (SD)	23.0 (16.9)	7.24 (9.76)	16.9 (16.5)
Median [Q1, Q3]	18.2 [9.94, 32.79]	3.87 [1.583, 8.559]	11.6 [4.03, 24.95]
Adiponectin, ng/mL*			
Mean (SD)	14,000 (11,500)	10,100 (8290)	13,000 (10,900)
Median [Q1, Q3]	10,472 [6685, 16,872]	7442 [5191, 11,805]	9645 [6164, 15,820]
C-peptide, ng/mL*			
Mean (SD)	2.47 (2.22)	2.40 (1.64)	2.45 (2.08)
Median [Q1, Q3]	2 [1.13, 3.23]	1.9 [1.161, 3.402]	1.98 [1.13, 3.29]
HbA1c, %*			
Mean (SD)	6.37 (1.48)	6.20 (1.72)	6.35 (1.51)
Median [Q1, Q3]	6 [5.6, 6.5]	5.8 [5.4, 6.2]	5.9 [5.6, 6.5]
Insulin, pmol/L*			
Mean (SD)	116 (113)	93.8 (117)	112 (114)
Median [Q1, Q3]	74.5 [48, 145]	67 [39, 106]	72 [48.0, 134.8]
Resistin, ng/mL*			
Mean (SD)	22.4 (26.6)	17.6 (15.0)	20.4 (22.6)
Median [Q1, Q3]	14.86 [9.13, 27.76]	12.24 [7.73, 23.80]	14.12 [8.37, 26.67]

Mean and median distribution of sample characteristics in women and men study participants

*ACE* adverse childhood experiences; *BMI* body mass index

*Indicates variables with missing values: income (2744), marital status (644), employment status (1333), menopause status (99), waist circumference (33,538), total cholesterol (35,805), HDL cholesterol (35,805), leptin (37,548), adiponectin (35,958), C-peptide (35,959), HbA1c (37,134), insulin (37,907), and resistin (38,020)

Across the cohort, 22,586 individuals (58.9%) experienced at least one ACE and median number of ACEs was 2 [Q1, Q3 = 1, 4]. Parental divorce was the most common ACE experienced among Black individuals and White men, followed by residing with an alcoholic or drug user. However, residing with an alcoholic or drug user was the most prevalent ACE for White women, followed by parental divorce (Table 2). Having a household member in prison was twice as prevalent in Black individuals compared to White, whereas emotional abuse and depression or mental illness in the household were more prevalent among White individuals (Table 2). Sexual abuse and emotional neglect were most prevalent in White women.

**Table 2:** Distribution of ACE components stratified by sex and race

ACE component	Black women (*N* = 16,408)	Black men (*N* = 7892)	White women (*N* = 9079)	White men (*N* = 4974)	Overall (*N* = 38,353)
Emotional abuse*	2613 (16.1%)	1165 (14.9%)	2415 (26.8%)	996 (20.2%)	7189 (18.9%)
Physical abuse*	2380 (14.6%)	1109 (14.2%)	1943 (21.5%)	845 (17.1%)	6277 (16.5%)
Sexual abuse*	2472 (15.2%)	550 (7.02%)	2147 (23.9%)	460 (9.31%)	5629 (14.8%)
Emotional neglect*	2947 (18.3%)	1046 (13.5%)	2247 (25.0%)	713 (14.4%)	6953 (18.4%)
Physical neglect*	1222 (7.56%)	644 (8.28%)	795 (8.86%)	341 (6.91%)	3002 (7.93%)
Parental divorce*	5708 (37.1%)	2819 (37.4%)	2507 (28.6%)	1205 (24.9%)	12,239 (33.5%)
Abused mother*	2087 (13.0%)	845 (10.8%)	1282 (14.3%)	491 (9.95%)	4705 (12.4%)
Alcoholic or drug user in household*	3479 (21.6%)	1581 (20.3%)	2637 (29.4%)	1123 (22.8%)	8820 (23.3%)
Depression/mental illness in household*	1891 (11.8%)	707 (9.10%)	1974 (22.0%)	655 (13.3%)	5227 (13.9%)
Incarcerated household member*	2124 (13.5%)	1243 (16.3%)	622 (7.02%)	328 (6.74%)	4317 (11.7%)

The distribution of number of sample participants for the different ACE components in Black women and men and White women and men as well as overall distribution of ACE components in the study

*ACE* adverse childhood experiences

*Indicates variables with missing values: emotional abuse (317), physical abuse (292), sexual abuse (364), emotional neglect (552), physical neglect (514), parental divorce (1806), abused mother (542), alcoholic or drug user in household (548), depression/mental illness in household (623), and incarcerated household member (1301)

### Association of ACEs with BMI

**Fig. 1:**
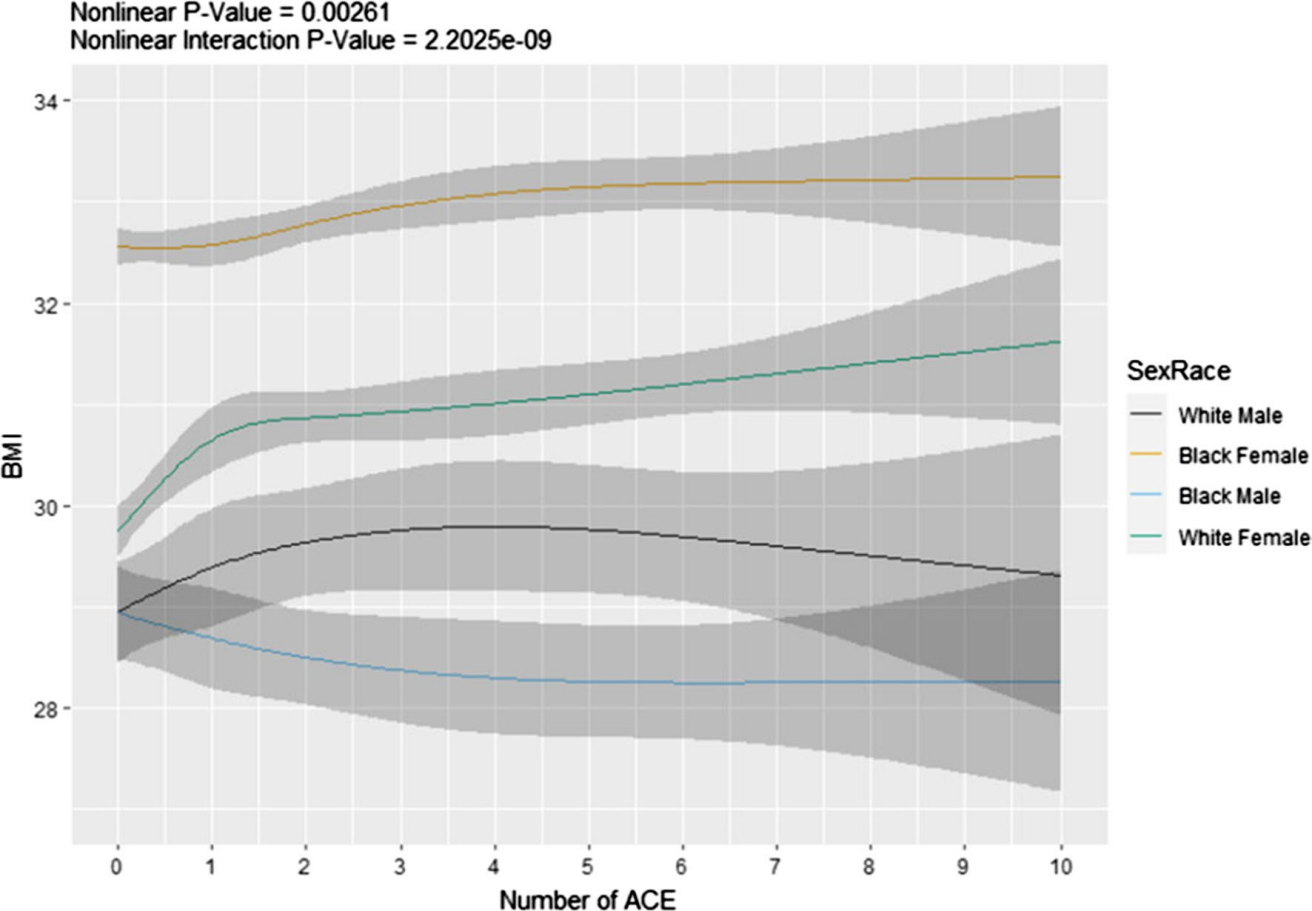
Predicted values for BMI versus ACE score stratified by sex and race. Regression analysis showing the effect of number of ACE on BMI in Black and White women and men. *ACE* adverse childhood experiences; *BMI* body mass index

There was a significant nonlinear association between total ACE score and BMI (*p*-value = 0.0026), which differed by both sex and race (interaction *p*-value < 0.0001), but not by menopause status in women. Figure 1 displays the association between ACE score and BMI by each combination of sex and race. Among Black women, BMI steadily increased with cumulative ACEs. One ACE event induced a steep increase in BMI in White women, which continued to steadily increase thereafter. In White men, BMI increased as ACEs increased from 0 to 3 but remained relatively stable among individuals with 4 or more ACEs. However, BMI decreased steadily with cumulative ACEs in Black men (Fig. 1).

Among Black women, emotional and sexual abuse were associated with higher BMI (Table 3). However, having an incarcerated family member was associated with lower BMI in both Black women and men. Physical abuse was associated with increased BMI in men regardless the race, while emotional neglect was specifically associated with increased BMI only in White women (Table 3).

**Table 3:** Association of ACE components with BMI

ACE component	Black women (*N* = 16,408)		Black men (*N* = 7892)		White women (*N* = 9079)		White men (*N* = 4974)	
	*β* estimate [95% CI]	*p* value	*β* estimate [95% CI]	*p* value	*β* estimate [95% CI]	*p* value	*β* estimate [95% CI]	*p* value
Emotional abuse	0.51 [0.03, 0.99]	0.037	0.01 [− 0.55, 0.57]	0.969	0.33 [− 0.25, 0.90]	0.268	0.17 [− 0.47, 0.80]	0.607
Physical abuse	0.17 [− 0.32, 0.66]	0.489	0.82 [0.026, 1.38]	0.004	0.54 [− 0.04, 1.11]	0.069	0.75 [0.12, 1.38]	0.020
Sexual abuse	0.89 [0.50, 1.28]	< 0.001	0.17 [− 0.47, 0.81]	0.595	0.18 [− 0.25, 0.61]	0.412	0.10 [− 0.53, 0.73]	0.753
Emotional neglect	0.16 [− 0.24, 0.55]	0.438	0.02 [− 0.50, 0.54]	0.933	0.60 [0.11, 1.09]	0.016	0.60 [− 0.02, 1.21]	0.058
Physical neglect	− 0.25 [− 0.77, 0.27]	0.354	− 0.09 [− 0.72, 0.54]	0.789	− 0.05 [− 0.71, 0.62]	0.894	0.53 [− 0.29, 1.35]	0.203
Parent divorce	− 0.04 [− 0.30, 0.22]	0.761	− 0.24 [− 0.55, 0.07]	0.129	0.35 [− 0.04, 0.74]	0.078	0.04 [− 0.39, 0.47]	0.843
Mother abused	− 0.28 [− 0.71, 0.14]	0.194	− 0.53 [− 1.09, 0.03]	0.064	0.28 [− 0.28, 0.84]	0.321	− 0.42 [− 1.13, 0.28]	0.238
Alcoholic or drug user in household	− 0.12 [− 0.46, 0.22]	0.495	− 0.21 [− 0.62, 0.21]	0.327	− 0.40 [− 0.81, 0.01]	0.056	0.14 [− 0.32, 0.60]	0.542
Depression/mental illness in household	0.02 [− 0.40, 0.44]	0.915	− 0.19 [− 0.78, 0.40]	0.519	− 0.39 [− 0.83, 0.05]	0.080	− 0.50 [− 1.05, 0.05]	0.075
Incarcerated household member	− 0.61 [− 0.99, − 0.23]	0.002	− 0.61 [− 1.04, − 0.18]	0.005	0.36 [− 0.31, 1.03]	0.292	− 0.39 [− 1.13, 0.35]	0.301

Results on the analysis of specific types of ACE on BMI in Black women and men and White women and men

*ACE* adverse childhood experiences

### Secondary analyses

**Fig. 2:**
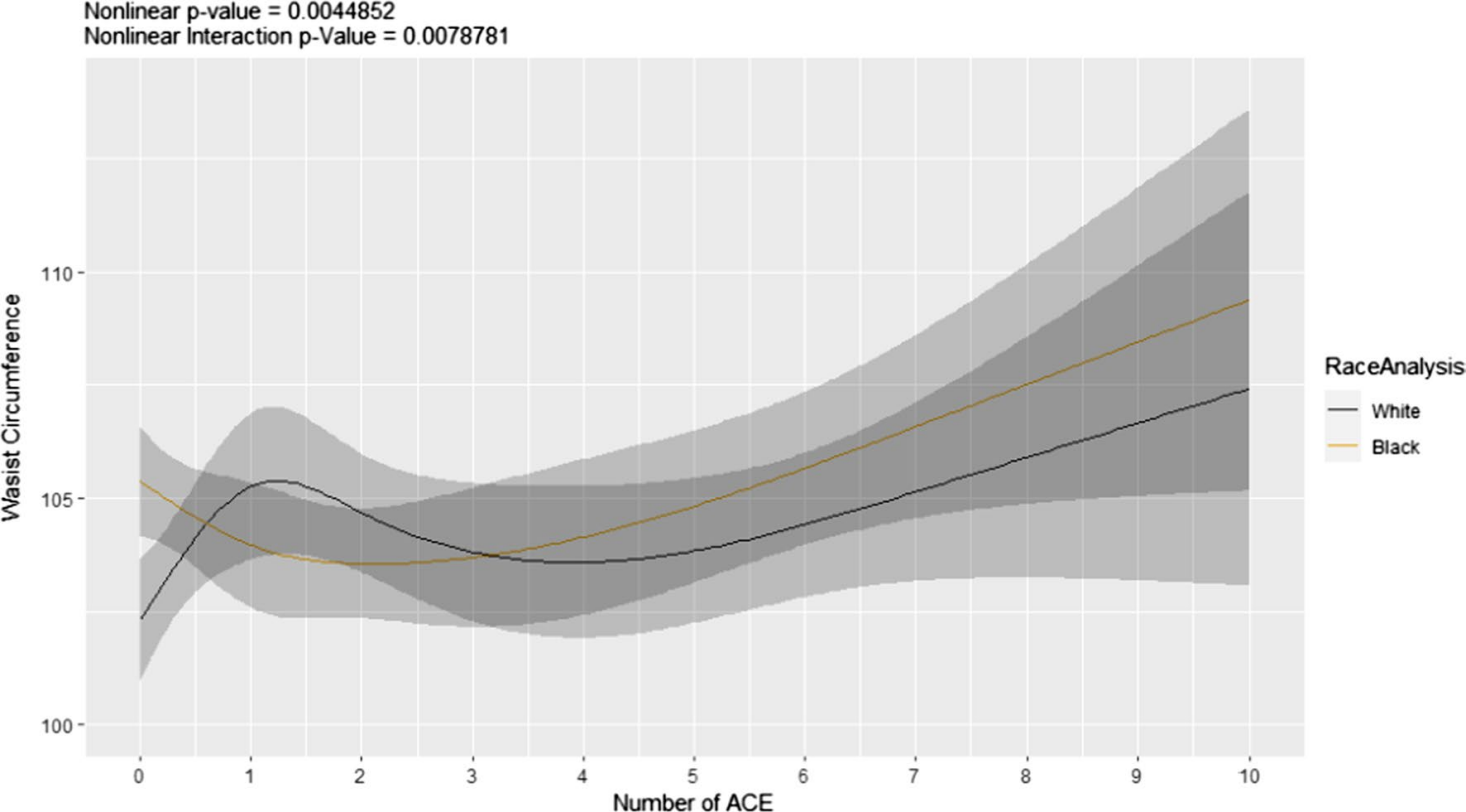
Predicted values for waist circumference versus ACEs by race. Regression analysis showing the effect of number of ACE on waist circumference (cm) in Black and White women and men. *ACE* adverse childhood experiences

The association between total ACE score with waist circumference was significantly nonlinear and differed by race (Fig. 2). In White individuals, waist circumference increased with 1 ACE event, decreased between 2 and 4 ACEs, and steadily increased in individuals with 5 or more ACEs. Notably, waist circumference in Black individuals progressively increased with 3 or more ACEs. Specifically, physical abuse was associated with a 4.85 cm increase in this measure (*p* < 0.001, Table 4.).

**Table 4:** Association of ACE components with waist circumference by race

ACE component	Black individuals (*N* = 2663)		White individuals (*N* = 2152)	
	*β* estimate [95% CI]	*p* value	*β* estimate [95% CI]	*p* value
Emotional abuse	1.10 [− 1.48, 3.68]	0.404	− 0.45 [− 3.21, 2.32]	0.751
Physical abuse	4.85 [2.20, 7.50]	< 0.001	1.96 [− 0.86, 4.78]	0.172
Sexual abuse	− 1.58 [− 4.05, 0.88]	0.207	− 0.78 [− 2.96, 1.40]	0.485
Emotional neglect	− 0.24 [− 2.47, 2.00]	0.836	2.19 [− 0.17, 4.54]	0.069
Physical neglect	0.30 [− 2.59, 3.19]	0.839	− 0.94 [− 4.03, 2.15]	0.552
Parent divorce	− 0.70 [− 2.19, 0.78]	0.353	− 0.92 [− 2.73, 0.89]	0.320
Mother abused	0.15 [− 2.40, 2.69]	0.910	1.24 [− 1.46, 3.95]	0.368
Alcoholic or drug user in household	− 1.53 [− 3.50, 0.44]	0.128	0.68 [− 1.28, 2.64]	0.495
Depression/mental illness in household	− 1.06 [− 3.59, 1.48]	0.413	− 0.94 [− 3.09, 1.21]	0.391
Incarcerated household member	− 1.49 [− 3.57, 0.58]	0.157	0.85 [− 2.04, 3.74]	0.564
*R*^2^/*R*^2^ adjusted	0.029/0.024		0.019/0.014	

Results on the analysis of the effect of specific types of ACE on waist circumference in Black women and White individuals. Adjusted for age, sex, and menopause status, 33.538 missing values

*ACE* adverse childhood experiences

**Fig. 3:**
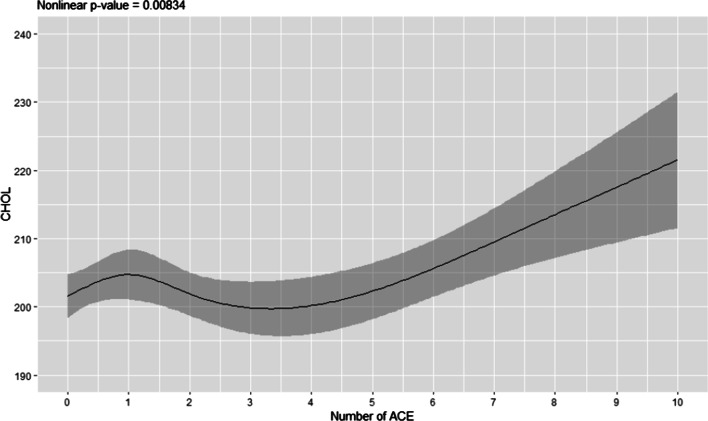
Predicted values for cholesterol versus ACEs. Regression analysis on the number of ACE on cholesterol in all sample participants. *ACE* adverse childhood experiences

The association of ACEs with total cholesterol was nonlinear. Total cholesterol increased from 0 to 1 ACE, decreased from 2–4 ACES, and increased with ACEs >4; this association was not modified by sex or race (Fig. 3). On average, maternal abuse during childhood increased 8.22 mg/dL in all groups combined (*p* = 0.007, Table 5).

**Table 5:** Association of ACE components with total cholesterol

ACE component	All participants (*N* = 2548)	
	*β* estimate [95% CI]	*p* value
Emotional abuse	1.06 [− 4.92, 7.04]	0.729
Physical abuse	− 0.85 [− 6.93, 5.23]	0.784
Sexual abuse	− 1.97 [− 7.25, 3.31]	0.464
Emotional neglect	− 2.00 [− 7.27, 3.27]	0.457
Physical neglect	1.76 [− 5.15, 8.68]	0.617
Parent divorce	2.53 [− 1.16, 6.22]	0.179
Mother abused	8.22 [2.21, 14.23]	0.007
Alcoholic or drug user in household	0.31 [− 4.20, 4.82]	0.893
Depression/mental illness in household	2.73 [− 2.50, 7.96]	0.306
Incarcerated household member	− 3.17 [− 8.59, 2.24]	0.251
*R*^2^/*R*^2^ adjusted	0.065/0.059	

Results on the analysis of specific types of ACE on cholesterol concentrations in all participants. Adjusted for sex, race, age, and menopause

*ACE* adverse childhood experiences

**Fig. 4:**
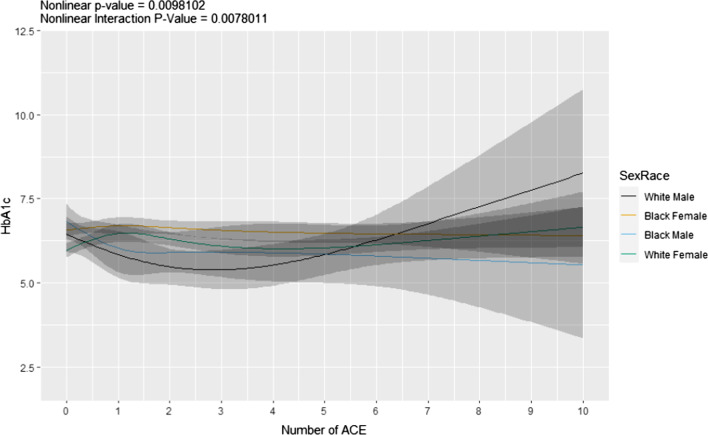
Predicted values for HbA1C versus ACE by sex and race. Regression analysis on the number of ACE on HbA1c in Black and White women and men. *ACE* adverse childhood experiences

While total ACE score was not associated with changes in C-peptide, parental divorce decreased it 0.24 ng/mL (*p* = 0.010, Additional file 1: Table S2). The association between ACEs and HbA1c was nonlinear and differed by sex and race. Among all groups, White men showed the lowest values from 1 to 4 ACEs and the highest values from 5 to 10 ACEs (Fig. 4). None of the individual ACE components were significantly associated with HbA1c in men, regardless of race (Additional file 1: Table S3). Emotional neglect was associated with decreased HbA1c in Black and White women (*p* = 0.004 and 0.006, respectively). However, emotional abuse and maternal abuse were associated with increased HbA1c in Black women (*p* = 0.025 and *p* = 0.026, respectively), while sexual abuse was associated with increased HbA1c only in White women (*p* = 0.014, Additional file 1: Table S3).

There was no association of total ACE score or individual ACE components with insulin, HDL cholesterol, leptin, adiponectin, or resistin (Additional file 1: Tables S4, S5).

## Discussion

The association between ACE and BMI has been reported previously by several cohorts, however, the composition of the SCCS is unique in terms of minority representation and includes many postmenopausal women. In addition, the current analysis provided the ability to pinpoint the direct contribution of specific adverse events on adult life’s metabolic health outcomes. These characteristics set this study apart from other similar reports that only evaluated the contribution of cumulative ACEs on health outcomes. Furthermore, we have investigated the effects of sex and race in the association between specific ACEs and metabolic health biomarkers. For example, emotional and sexual abuse is associated with higher BMI in Black women whereas physical abuse was associated with increased BMI in Black and White men. However, cumulative ACEs were associated with decreased BMI in Black men, alluding to potential different mechanism for the effect of individual and combined stressors. Taken together, the outcomes of this study suggest that sex and race, but not menopausal status in women, influence the body adiposity and metabolic health biomarkers in individuals exposed to ACEs.

Previous publications using data from the SCCS cohort have reported associations between ACE, low-income status, and cancer [30, 31]. Mouton et al. have shown that ACEs were related with an increased risk of developing morbid obesity in White individuals regardless of sex. In women only, regardless of race, ACEs increased high-risk cancer-causing behaviors (i.e., smoking) and decreased cancer screening measures. In the current study, we focused on the association between ACEs (combined and individual) and BMI and a cluster of metabolic health biomarkers available at the SCCS. Our cross-sectional analysis showed that Black women in our sample possessed the highest BMI values compared to Black men and White subjects, showing that cumulative ACE correlated positively with BMI.

While BMI is a useful measurement that provides a rapid estimate of body adiposity, it is now accepted that there are racial and ethnic differences in the relationship between BMI and adiposity, and the cutoff values determined for BMI are based on sex and race [32, 33]. In addition, increased central adiposity and waist circumference can serve as a predictor for morbidity, independent of BMI [34, 35]. Katzmarzyk et al. showed that increased waist circumference strongly correlates with metabolic syndrome [36]; however, a large proportion of men with normal waist circumference have multiple risk factors for metabolic disease and an increased risk of mortality. On the other hand, individuals considered to have a normal BMI are still at increased risk of metabolic disease if weight is mostly located around the waist [37, 38]. Due to the link between increased waist circumference and metabolic health risks, this data is in line with findings from Moore et al. showing that among all diagnosed cases of individuals with metabolic syndrome, non-Hispanic Black adults had the higher prevalence of metabolic syndrome compared to others diagnosed [39]. Noteworthy, study investigating the sympathetic overactivity in Black subjects reported that lean and overweight Black men display 20–40% increased muscle nerve activity compared to Black and White women and White men, which was dissociated from fat mass [40]. Therefore, Black men show adiposity-independent sympathetic overactivity. The negative association we have found between ACEs and BMI only in Black men suggests that these individuals could have a further increase in sympathetic tone, leading to greater energy expenditure and reduced fat mass. Moreover, physical abuse was associated with a 4.9 cm increase in waist circumference in Black individuals, which combined with reduced BMI suggests the accumulation of central adiposity in Black men exposed to this particular ACE and increased metabolic risk.

There is a paucity of studies investigating sex differences in CVD and stress-related comorbidities through life’s trajectory. There is substantial evidence pointing to a range of psychosocial disadvantages that predominantly affect women’s physiological responses to stress, such as access to health, intrafamily conflict, caregiving demands, and financial hardship. Women at high risk may include single mothers with low socioeconomic status, working mothers, and older women living alone with limited social support [41–43]. Noteworthy, the importance to address how psychosocial factors increase the risk for metabolic disease and other chronic diseases is the impact on future generation health status [44–46]. Based on the rising number of studies contemplating the effect of sex in the interplay with environmental stressors, there is a hopeful trend to incorporate this matter into future research. Long-lasting effects of emotional stress could serve as a silent causal factor for the uncontrolled epidemic rates of T2D and ischemic heart disease. Identification of ACEs and the specific types of ACEs affecting women may help to improve obesity prevention and reduce health disparities. For instance, increasing awareness in the medical community of issues related to women with obesity, improving obesity and cardiovascular disease diagnosis, and advancing research in women’s health. In addition, given that women remain underrepresented in clinical trials, investigators are often seeking sites on women’s cardiovascular programs to increase recruitment. Women show poor management and outcomes resulting from the therapeutic management of cardiovascular and metabolic diseases, thus, targeted programs are essential to correct the persistent inequities of weight loss management and cardiovascular disease care and prevention in women [47].

One limitation of this study is the self-reported approach to collect data. Flegal et al. compared the results of three self-reported height and weight data from the National Health and Nutrition Examination Survey (NHANES), the National Health Interview Survey, and the Behavioral Risk Factor Surveillance System for the years 1999–2016. In all surveys, mean BMI from self-reported data was lower than mean BMI from measured data. Also, the distribution of BMI was narrower for self-reported than for measured data, leading to lower estimates of obesity prevalence. Furthermore, mean self-reported weight, BMI, and obesity prevalence in the National Health Interview Survey and Behavioral Risk Factor Surveillance System were lower than self-report in NHANES for women. This data suggest that the age and race composition of each cohort may influence the correlation between self-reported and measured data. Taken together, self-reported data tend to underestimate BMI compared to measured data, indicating that the effects of ACE on BMI analyzed in the SCCS cohort could be even greater, particularly in women.

Of note, another limitation of this study is the lack of power for secondary analysis. While a positive aspect of this cohort design is the large percentage of Black individuals and women participants. One drawback is the lack of White individuals and male data to properly compare groups in secondary analysis. The unequal composition of groups does not allow for adequate analysis and for more meaningful conclusions. Thus, this study misses out on the opportunity for possible direct connections with effect of sex and race on the modulation of ACE, BMI, and metabolic biomarkers. Therefore, future study designs should account for these differences in order to achieve outcomes providing further insights for potential interventional studies.

Chronic stress is most strongly associated with A1c, particularly among subgroups that face disproportionate exposure to stress, such as minority groups or adolescents/young adults. Mechanisms linking stress with increased A1c association physiological, psychological, behavioral, and environmental components. Therefore, the potential cause for the negative association with emotional neglect remain elusive in certain individuals. Additional studies that are sufficiently powered are needed to better understand the effect of metabolic biomarkers on ACEs in an aged population.

In conclusion, ACEs are associated with BMI in women, but several ACE components display an unequal and unique contribution to this effect that varies based on sex and race. Family resilience could be used as a coping mechanism to augment the negative impacts of ACE but only in Black and Hispanic children. Along these lines, it has been reported that while White children came from more socioeconomically wealthy backgrounds, they performed more poorly at higher ACEs compared to Black and Hispanic children [48]. Similarly, our study reports differences in response to ACEs across race/ethnicity and supports the notion that both of these factors should be considered when evaluating patient health, therapeutic interventions, and disease trajectory outcomes. As healthcare professionals and researchers come together to reduce the obesity epidemic, we are facing limitations due to factors such as (1) genetic and biological risk factors; (2) individual behaviors (parenting styles, dietary patterns, physical activity levels, medication use, sleep, stress management); and (3) psychosocial factors that determine access to health care, nutrition, physical activity and the well-being of men and women, particularly affecting the first decade of life.

## Clinical perspectives

If current trends in obesity persist, then by 2030 approximately half of the population will be considered obese. The impact of ACE on obesity and cardiometabolic risk is well established; however, this study highlights that the effect of cumulative ACEs as well as specific adverse experiences seem to trigger the alteration of metabolic biomarkers with a unique signature depending on sex or race. Further investigation of these associations is critical to develop a more effective preventive and personalized medicine.

Understanding the contribution of non-traditional risk factors to the development of obesity and metabolic disease may provide a valuable tool to fight health disparities and reduce the projected rates of these chronic diseases.

## Supplementary Information

**Additional file 1.** ACE Questionnaire to self-report ACEs. **Table S1.** Secondary analysis group composition. **Table S2.** Association of ACE components with C-peptide. **Table S3.** Association of ACE components with HbA1c. **Table S4.** Association of total ACE with insulin. **Table S5.** Association of ACE components with insulin.

## Data Availability

Please contact author for data requests.

## Abbreviations

SCCS: Southern Community Cohort Study
ACE: Adverse childhood experience
BMI: Body mass index
T2D: Type 2 diabetes
