# Supplementary material for: Sex and race define the effects of adverse childhood experiences on self-reported BMI and metabolic health biomarkers
Source: Biol Sex Differ. 2022 Jun 15;13:29. doi: 10.1186/s13293-022-00439-x (PMC9202152; doi:10.1186/s13293-022-00439-x)
Supplement: Supplementary file 1 — Additional file 1. ACE Questionnaire to self-report ACEs. Table S1. Secondary analysis group composition. Table S2. Association of ACE components with C-peptide. Table S3. Association of ACE components with HbA1c. Table S4. Association of total ACE with insulin. Table S5. Association of ACE components with insulin. [file 13293_2022_439_MOESM1_ESM.docx]

**Sex and Race define the Effect of Adverse Childhood Experiences on Self-reported BMI and metabolic health biomarkers**

**Additional Material**

Jacqueline Leachman^1^, Kory Heier^2^, Feitong Lei^2^, Nermin Ahmed^1^, Carolina Dalmasso, S. Duncan^2^, and Analia S. Loria^1^

1 Department of Pharmacology and Nutritional Sciences, University of Kentucky, Lexington KY

2 Department of Biostatistics, University of Kentucky, Lexington KY

**ACE Questionnaire to self-report ACEs.**

When you were growing up, during your first 18 years of life:

- did a parent or other adult in the household often swear at you, insult you, put you down, or humiliate you or act in a way that made you afraid that you might be physically hurt? (emotional abuse)
- did a parent or other adult in the household often push, grab, slap, or throw something at you or ever hit you so hard that you had marks or were injured? (physical abuse)
- did an adult or person at least 5 years older than you ever touch or fondle you or have you touch their body in a sexual way or try to or actually have oral, anal, or vaginal sex with you? (sexual abuse)
- did you often feel that no one in your family loved you or thought you were important or special or your family didn't look out for each other, feel close to each other, or support each other? (emotional neglect)
- did you often feel that you didn't have enough to eat, had to wear dirty clothes, and had no one to protect you or your parents were too drunk or high to take care of you or take you to the doctor if you needed it? (physical neglect)
- were your parents ever separated or divorced? (parental divorce)
- was your mother or stepmother often pushed, grabbed, slapped, or had something thrown at her or sometimes or often kicked, bitten, hit with a fist, or hit with something hard or ever repeatedly hit over at least a few minutes or threatened with a gun or a knife? (abused mother)
- did you live with anyone who was a problem drinker or alcoholic or who used street drugs? (alcoholic or drug user in household)
- was a household member depressed or mentally ill or did a household member attempt suicide? (depression/mental illness in household)
- did a household member go to prison? (incarcerated household member)

**Additional Table S1.** Secondary Analysis Group Composition

|  | Black Women | White Women | Black Men | White Men | Total |
| --- | --- | --- | --- | --- | --- |
| Waist Circumference | 1661 | 1544 | 1002 | 608 | 4815 |
| Total Cholesterol | 1239 | 695 | 441 | 173 | 2548 |
| HDL | 1239 | 695 | 441 | 173 | 2548 |
| Leptin | 268 | 227 | 200 | 110 | 805 |
| Adiponectin | 1025 | 762 | 440 | 168 | 2395 |
| leptin/adiponectin | 265 | 226 | 199 | 110 | 800 |
| C-Peptide | 1025 | 761 | 441 | 167 | 2394 |
| HbA1c | 526 | 547 | 66 | 80 | 1219 |
| Insulin | 360 | 0 | 86 | 0 | 446 |
| Resistin | 98 | 96 | 61 | 78 | 333 |

***Additional Table S1. Secondary Analysis Group Composition*.** Number of samples for different secondary analysis for Black and White Women and Men.

**Additional Table S2.** Association of ACE components with C-Peptide

|  | **All Participants**  **(N=2,394)** | |
| --- | --- | --- |
| **ACE Component** | *β Estimate*  *[95% CI]* | *p-value* |
| Emotional Abuse | 0.17  [-0.14, 0.47] | 0.277 |
| Physical Abuse | -0.04  [-0.35, 0.26] | 0.789 |
| Sexual Abuse | 0.17  [-0.10, 0.44] | 0.226 |
| Emotional Neglect | -0.02  [-0.29, 0.25] | 0.878 |
| Physical Neglect | -0.07  [-0.42, 0.29] | 0.709 |
| Parent Divorce | -0.24  [-0.43, -0.06] | 0.010 |
| Mother Abused | -0.15  [-0.46, 0.15] | 0.328 |
| Alcoholic or drug user in household | -0.03  [-0.25, 0.20] | 0.820 |
| Incarcerated household member | -0.06  [-0.34, 0.22] | 0.686 |
| R^2^ / R^2^ Adjusted | 0.018 / 0.012 | |

***Additional Table S2. Association of ACE components with C-Peptide*.** Results on the analysis of specific types of ACE on C-Peptide in all study participants. ACE, Adverse Childhood Experiences. Adjusted for sex, race, age, and menopause, 35,959 missing values.

**Additional Table S3.** Association of ACE components with HbA1c

|  | Black Men  (N=66) | |  | White Men  (N=80) | |  | Black Women  (N=526) | |  | White Women  (N=547) | |
| --- | --- | --- | --- | --- | --- | --- | --- | --- | --- | --- | --- |
| ACE Component | *β Estimate*  *[95% CI]* | *p-value* |  | *β Estimate [95% CI]* | *p-value* |  | *β Estimate*  *[95% CI]* | *p-value* |  | *β Estimate*  *[95% CI]* | *p-value* |
| Emotional Abuse | -0.67  [-3.33, 1.98] | 0.614 |  | -0.53  [-1.72, 0.67] | 0.382 |  | 0.58  [0.07, 1.08] | 0.025 |  | -0.00  [-0.36, 0.35] | 0.995 |
| Physical Abuse | 1.07  [-2.82, 4.96] | 0.583 |  | 0.43  [-0.72, 1.58] | 0.457 |  | -0.42  [-0.95, 0.10] | 0.115 |  | 0.19  [-0.15, 0.53] | 0.275 |
| Sexual Abuse | 1.00  [-3.07, 5.07] | 0.623 |  | -0.26  [-1.28, 0.76] | 0.611 |  | -0.25  [-0.70, 0.21] | 0.284 |  | 0.37  [0.08, 0.66] | 0.014 |
| Emotional Neglect | -0.51  [-2.85, 1.82] | 0.662 |  | 0.19  [-0.82, 1.20] | 0.712 |  | -0.66  [-1.12, -0.21] | 0.004 |  | -0.43  [-0.74, -0.13] | 0.006 |
| Physical Neglect | -0.27  [-2.38, 1.84] | 0.798 |  | -1.02  [-2.83, 0.78] | 0.260 |  | 0.41  [-0.23, 1.05] | 0.210 |  | 0.16  [-0.27, 0.60] | 0.465 |
| Parent Divorce | -0.47  [-1.75, 0.81] | 0.466 |  | -0.71  [-1.54, 0.12] | 0.094 |  | 0.11  [-0.18, 0.40] | 0.473 |  | 0.15  [-0.11, 0.40] | 0.263 |
| Mother Abused | -0.81  [-5.20, 3.59] | 0.715 |  | 0.89  [-0.27, 2.04] | 0.130 |  | 0.56  [0.07, 1.04] | 0.026 |  | -0.03  [-0.40, 0.33] | 0.858 |
| Alcoholic or drug user in household | -0.87  [-2.84, 1.10] | 0.380 |  | -0.20  [-1.12, 0.71] | 0.659 |  | -0.19  [-0.57, 0.18] | 0.309 |  | 0.00  [-0.27, 0.27] | 0.990 |
| Depression/mental illness in household | 0.35  [-2.50, 3.19] | 0.808 |  | -0.12  [-1.00, 0.75] | 0.778 |  | 0.04  [-0.42, 0.50] | 0.856 |  | -0.20  [-0.51, 0.11] | 0.204 |
| Incarcerated household member | 0.06  [-1.99, 2.10] | 0.956 |  | -0.25  [-1.22, 0.72] | 0.611 |  | -0.20  [-0.62, 0.22] | 0.345 |  | 0.22  [-0.18, 0.62] | 0.277 |
| R^2^ / R^2^ Adjusted | 0.070 / -0.119 | |  | 0.089 / -0.058 | |  | 0.073 / 0.051 | |  | 0.034 / 0.012 | |

***Additional Table S3. Association of ACE components with HbA1c*.** Results on the analysis of specific types of ACE on HbA1c in Black and White Women and Men. ACE, Adverse Childhood Experiences. Models additionally adjusted for age and menopause status (women only). 37,134 missing values.

**Additional Table S4.** Association of Total ACE with Insulin

| **Dependent Variable** | **β Estimate for effect of ACEs**  **[95% CI]** | **p-value** |
| --- | --- | --- |
| Insulin | 0.26  [-1.43, 0.91] | 0.66 |
|  | | |

**Additional Table S4. Association of Total ACE with Insulin.** Results on the analysis of total ACE on Insulin levels in all study participants. ACE, Adverse Childhood Experiences. Models adjusted for age, sex, race, and menopause status.

**Additional Table S5.** Association of ACE components with Insulin

|  | **All Participants**  **(N=446)** | |
| --- | --- | --- |
| **ACE Component** | *β Estimate*  *[95% CI]* | *p-value* |
| Emotional Abuse | 19.80  [-17.38, 56.97] | 0.296 |
| Physical Abuse | -24.71  [-64.86, 15.44] | 0.227 |
| Sexual Abuse | 14.23  [-22.25, 50.71] | 0.444 |
| Emotional Neglect | -0.50  [-34.13, 33.13] | 0.977 |
| Physical Neglect | -36.59  [-80.60, 7.41] | 0.103 |
| Parent Divorce | -24.91  [-47.75, -2.07] | 0.033 |
| Mother Abused | -14.25  [-53.31, 24.81] | 0.474 |
| Alcoholic or drug user in household | 9.76  [-20.02, 39.55] | 0.520 |
| Depression/mental illness in household | 21.48  [-13.88, 56.84] | 0.233 |
| Incarcerated household member | -23.01  [-56.55, 10.53] | 0.178 |
| R^2^ / R^2^ Adjusted | 0.049 / 0.020 | |
|  | | |

**Additional Table S5. Association of ACE components with Insulin**. Results on the analysis of individual ACE components on Insulin levels in all study participants. ACE, Adverse Childhood Experiences. Models adjusted for age, sex, race, and menopause status. 37,907 missing values
